# Suppression of Superoxide-Hydrogen Peroxide Production at Site I_Q_ of Mitochondrial Complex I Attenuates Myocardial Stunning and Improves Postcardiac Arrest Outcomes

**DOI:** 10.1097/CCM.0000000000004095

**Published:** 2020-01-15

**Authors:** Lin Piao, Yong-Hu Fang, Robert B. Hamanaka, Gökhan M. Mutlu, Cameron Dezfulian, Stephen L. Archer, Willard W. Sharp

**Affiliations:** 1 Section of Emergency Medicine, Department of Medicine, University of Chicago, Chicago, IL.; 2 Section of Pulmonary and Critical Care Medicine, Department of Medicine, University of Chicago, Chicago, IL.; 3 Safar Center for Resuscitation Research, Critical Care Medicine Department, University of Pittsburgh School of Medicine, Pittsburgh, PA.; 4 Department of Medicine, Queen’s University, Kingston, ON, Canada.

**Keywords:** cardiopulmonary resuscitation, heart arrest, metabolism, mitochondria, oxygen consumption, reactive oxygen species

## Abstract

Cardiogenic shock following cardiopulmonary resuscitation for sudden cardiac arrest is common, occurring even in the absence of acute coronary artery occlusion, and contributes to high rates of postcardiopulmonary resuscitation mortality. The pathophysiology of this shock is unclear, and effective therapies for improving clinical outcomes are lacking.

**Design:**

Laboratory investigation.

**Setting:**

University laboratory.

**Subjects:**

C57BL/6 adult female mice.

**Interventions:**

Anesthetized and ventilated adult female C57BL/6 wild-type mice underwent a 4, 8, 12, or 16-minute potassium chloride-induced cardiac arrest followed by 90 seconds of cardiopulmonary resuscitation. Mice were then blindly randomized to a single IV injection of vehicle (phosphate-buffered saline) or suppressor of site I_Q_ electron leak, an inhibitor of superoxide production by complex I of the mitochondrial electron transport chain. Suppressor of site I_Q_ electron leak and vehicle were administered during cardiopulmonary resuscitation.

**Measurements and Main Results:**

Using a murine model of asystolic cardiac arrest, we discovered that duration of cardiac arrest prior to cardiopulmonary resuscitation determined postresuscitation success rates, degree of neurologic injury, and severity of myocardial dysfunction. Post-cardiopulmonary resuscitation cardiac dysfunction was not associated with myocardial necrosis, apoptosis, inflammation, or mitochondrial permeability transition pore opening. Furthermore, left ventricular function recovered within 72 hours of cardiopulmonary resuscitation, indicative of myocardial stunning. Postcardiopulmonary resuscitation, the myocardium exhibited increased reactive oxygen species and evidence of mitochondrial injury, specifically reperfusion-induced reactive oxygen species generation at electron transport chain complex I. Suppressor of site I_Q_ electron leak, which inhibits complex I-dependent reactive oxygen species generation by suppression of site I_Q_ electron leak, decreased myocardial reactive oxygen species generation and improved postcardiopulmonary resuscitation myocardial function, neurologic outcomes, and survival.

**Conclusions:**

The severity of cardiogenic shock following asystolic cardiac arrest is dependent on the length of cardiac arrest prior to cardiopulmonary resuscitation and is mediated by myocardial stunning resulting from mitochondrial electron transport chain complex I dysfunction. A novel pharmacologic agent targeting this mechanism, suppressor of site I_Q_ electron leak, represents a potential, practical therapy for improving sudden cardiac arrest resuscitation outcomes.

## Abstract 2

Supplemental Digital Content is available in the text.

Sudden cardiac arrest (CA) both in and out of hospital is common and has high morbidity and mortality (1). Early, high-quality cardiopulmonary resuscitation (CPR) has been demonstrated to increase survival, but its effectiveness deteriorates within minutes if its initiation is delayed (2, 3). Delayed CPR is common and often associated with cardiogenic shock resulting in hemodynamic instability and poor neurologic outcomes (4). The severity of post-CPR shock may also contribute to the extent of neurologic outcomes in surviving patients (5). Post-CPR cardiogenic shock occurs even in the absence of acute coronary artery occlusion and is a component of the “post-CA syndrome” (5, 6). The pathophysiology of post-CPR cardiogenic shock is unknown, and effective therapies are lacking.

Myocardial ischemia of short duration followed by adequate coronary flow restoration results in reversible myocardial dysfunction without necrosis. This cardiac pathology is termed “myocardial stunning” (7, 8). It was originally used to describe regional non–infarcted ventricular wall movement abnormalities, following brief coronary artery occlusion/reperfusion injuries, but has since been used to describe patients experiencing cardiogenic shock after percutaneous coronary artery intervention and cardiopulmonary bypass surgery (9, 10). Myocardial stunning is not commonly recognized as mediating post-CPR shock, and although it has been described in the setting of ventricular fibrillation–induced CA (8, 11), it has not been studied in other forms of CA, such as asystolic CA. Furthermore, the molecular mechanisms mediating myocardial stunning are unknown because it has been described as “lacking clinical relevance” (9).

Myocardial mitochondria occupy one third of the heart’s volume and are central regulators of calcium, reactive oxygen species (ROS), and metabolism. Mitochondria are dynamic organelles undergoing regulated fusion (joining) and fission (dividing) events (12, 13). Our group was the first to demonstrate evidence of mitochondrial fission following CA, its mediation of myocardial dysfunction through fission-induced ROS generation (14). In addition to mitochondrial fission, the accumulation of succinate during cellular ischemia results in increased electron leak and generation of superoxide and/or hydrogen peroxide (H_2_O_2_) (15, 16). Limiting electron leak and ROS generation using inhibitors of mitochondrial electron transport during ischemia/reperfusion (IR) have shown promise, but their utility is limited by their negative effects on metabolism (17–19).

Recently, Brand et al (16) have identified compounds that protect against H_2_O_2_ production induced by electron leak at sites I_Q_ (the ubiquinone-binding site of complex I, the active site during reverse electron transport), II_F_ (the flavin site of complex II), or III_Q0_ (the outer ubiquinone-binding site of complex III) in isolated skeletal muscle. One compound, the suppressor of site I_Q_ electron leak (S1QEL), limited ROS generation at complex I without affecting normal electron transport. S1QEL also attenuated oxidative damage in several cell types while limiting infarct size in Langendorff-perfused mouse hearts. In this study, we hypothesized that the severity of cardiogenic shock following CPR is dependent on the length of CA and by virtue of lacking myocardial necrosis, is reversible, consistent with myocardial stunning. Furthermore, we hypothesized that myocardial stunning is mediated by increased mitochondrial complex I ROS generation and that its therapeutic targeting by S1QEL could improve postresuscitation outcomes. Findings in this study support the hypothesis and suggest that S1QEL has potential as a therapeutic agent to improve outcomes in CA.

## MATERIAL AND METHODS

### CA Mice Model

Asystolic CA was induced in adult (age, 6–8 mo; 20–30 g) retired breeder female C57BL/6 mice as previously described (14). Briefly, mice were anesthetized (3% vaporized isoflurane) and instrumented. Asystolic CA was induced by 0.08 mg/g potassium chloride injection via the jugular vein. Following 12 minutes of CA, the ventilator was reconnected and manual chest compression was performed at a rate of 350~400 beats/min. After 90 seconds of CPR, 1.5 μg of epinephrine was injected. The CA protocol used in this study is illustrated in **Supplemental Figure 1** (Supplemental Digital Content 1, http://links.lww.com/CCM/F61). In accordance with National Institutes of Health guidelines, the University of Chicago Institutional Animal Care and Use Committee approved all animal procedures. In total, 121 mice entered the study. Twenty two mice died due to surgical failure and 49 mice could not be resuscitated. Additional details are described in the **supplemental methods** (Supplemental Digital Content 1, http://links.lww.com/CCM/F61).

#### Neurologic Scoring of Animals.

Neurologic deficits after CA (2, 6, 24, 48, and 72 hr) in mice were determined using a 12-point mouse neurologic scoring system (20). Scores ranged from 0 (no response or worst) to 2 (normal) along six domains: paw pinch, righting reflex, breathing, spontaneous movement, motor-global, and motor-focal. The scores for each of the six domains were determined in a blinded fashion and summed to achieve the neurologic score.

#### Mitochondria Isolation.

Mitochondria were obtained from post-CA hearts as previously described (13). Briefly, hearts from Sham and post-CA mice were collected at 15 minutes after CPR, then minced and incubated with trypsin before homogenization with a glass/teflon Potter Elvehjem homogenizer (Fisher Scientific, Hanover Park, IL). Heart homogenates were centrifuged at 800*g* × 5 minutes at 4°C and the supernatant collected and centrifuged at 8,000*g* × 5 minutes at 4°C twice to obtain purified cardiac mitochondria. See the supplemental methods (Supplemental Digital Content 1, http://links.lww.com/CCM/F61) for further details.

### Mitochondrial Permeability Transition Pore Opening

Mitochondrial permeability transition pore (mPTP) opening induced by calcium was determined in freshly isolated cardiac mitochondria (13). The absorbance was continuously measured using a Cytation 3 (BioTek, Winooski, VT) 96-well plate reader at 540 nm. Additional details are described in the supplemental methods (Supplemental Digital Content 1, http://links.lww.com/CCM/F61).

### Complex I Enzyme Activity

Complex I activity was measured using an enzyme activity dipstick assay (Abcam, Cambridge, MA) following the manufacturer protocol. In principle, immunocaptured Complex I oxidizes nicotinamide adenine dinucleotide, reduced form (NADH) and the resulting hydrogen ion (H+) reduces nitrotetrazolium blue (NBT) to form a blue-purple precipitate at the complex I antibody line on the dipstick immersed in complex I activity buffer containing NADH (substrate) and NBT (electron acceptor). The signal intensity of this precipitate corresponds to the level of complex I enzyme activity (blue band) in the sample. The intensity was analyzed by using Fiji 6 (National Institutes of Health, Bethesda, MD).

### Superoxide-H_2_O_2_ Production in Cardiac Mitochondria

To induce H_2_O_2_ production from site I_Q_ in cardiac mitochondria, 20-mM glycerol 3-phosphate was added to isolated mitochondria (1 μg/100 μL) in respiration medium with 50-μM Amplex Red and 2-mU/mL horseradish peroxidase (ThermoFisher, Waltham, MA) (16). Fluorescence was monitored using a microplate reader (SpectraMax iD3; Molecular Devices, Sunnyvale, CA) for excitation at 540 nm and emission detection at 590 nm at 37°C after 30 minutes incubation.

### Seahorse Measurement of Mitochondrial Oxygen Consumption Rates

Isolated mitochondria (1 μg/100 μL) from the hearts of Sham and post-CPR mice were suspended in 24-well plates. Oxygen consumption rates (OCRs) were determined using the Seahorse XF24 Extracellular Flux Analyzer (Seahorse Bioscience, Billerica, MA), as previously described (21). Complex I OCR was measured using the substrates 10-mM pyruvate + 2-mM malate. Complex II OCR was measured using the substrate 10-mM succinate and an inhibitor of reverse electron flow, 2-µM rotenone. Additional details are described in the supplemental methods (Supplemental Digital Content 1, http://links.lww.com/CCM/F61).

### Statistics

Comparisons between groups containing normally distributed data were made using analysis of variance with Tukey test or the Student *t* test. Mann-Whitney *U* test and Kruskal-Wallis test were applied for nonparametric statistics. The survival curves were compared using a log rank (Mantel Cox) test. Analysis was performed using Prism software (Graph Pad, La Jolla, CA). Data were presented as mean ± sem. Values of *p* less than 0.05 were considered statistically significant.

### Supplemental Methods

Details regarding mouse echocardiography and different staining methods are provided in the supplemental methods (Supplemental Digital Content 1, http://links.lww.com/CCM/F61).

## RESULTS

### CA Duration Determines Post-CPR Myocardial Dysfunction and Resuscitation Outcomes

**Figure 1.**
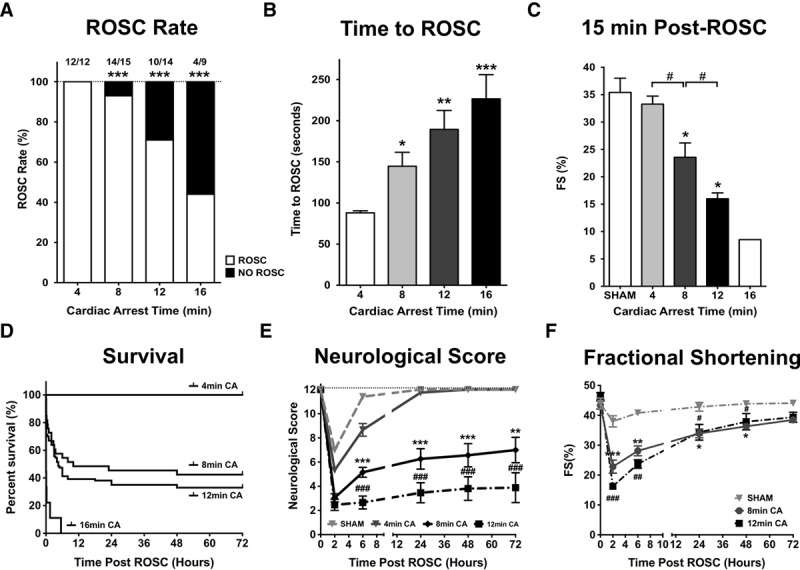
Duration of cardiac arrest determines postcardiopulmonary resuscitation (CPR) outcomes. **A**, Return of spontaneous circulation (ROSC) rates following 4, 8, 12, and 16 min of cardiac arrest (CA). **B**, Time of CPR to achieve ROSC. *n* = 12, 15, 14, and 9, respectively. *p < 0.05; **p < 0.01; ***p < 0.001 versus 4-min group. **C**, Percent left ventricular fractional shortening 15 min after achieving ROSC for different durations of CA. *n* = 17, 12, 12, 12, and 1, respectively. **D**, Kaplan-Meyer Curve demonstrating survival following different durations of CA. *n* = 22, 28, 34, and 9, respectively. **E**, Neurologic scores following CA of increasing duration. *n* = 12, respectively. **F**, Percent left ventricular fractional shortening recovery over time following CA. *n* = 7, 7, and 9, respectively. *p < 0.05; **p < 0.01; ***p < 0.001 versus sham. #*p* < 0.05.

Using our previously established model of induced asystolic CA, we investigated the effects of cardiac duration on resuscitation outcomes (14). Baseline characteristics of the mice and CPR quality were recorded (**Supplemental Table 1**, Supplemental Digital Content 1, http://links.lww.com/CCM/F61). Increasing the durations of CA reduced rates of return to spontaneous circulation (ROSC) and increased the CPR time needed to achieve ROSC (ROSC rate: 100%, 93%, 71%, and 44% in 4, 8, 12, and 16-min group, respectively; time to ROSC: 88 ± 2, 145 ± 17, 189 ± 23, and 227 ± 29 min, respectively; **Fig. 1**, ***A*** and ***B***). Post-CPR myocardial dysfunction was proportional to the duration of CA (fractional shortening at 15 min post-ROSC: 35% ± 2%, 33% ± 1%, 24% ± 3%, 16% ± 1%, and 9% in sham, 4, 8, 12, and 16-min group, respectively; **Fig. 1*C***) and predicted survival 2 hours after ROSC (**Fig. 1*D***). CA duration also correlated strongly with the severity of neurologic injury over 72 hours after ROSC (**Fig. 1*E***). Despite being severely depressed during the first 6 hours following ROSC, myocardial function gradually improved to near-baseline measurements over the following 72 hours (fractional shortening at 72 hr post-ROSC: 41% ± 1% in sham, 39% ± 2% in 12-min group; **Fig. 1*F***). These data are consistent with clinical observations, which show that post-CPR outcomes worsen as a function of CA duration (2, 6) and that post-CPR myocardial dysfunction recovers over time (22).

### Post-CPR Myocardial Dysfunction Is Consistent With Myocardial Stunning

**Figure 2.**
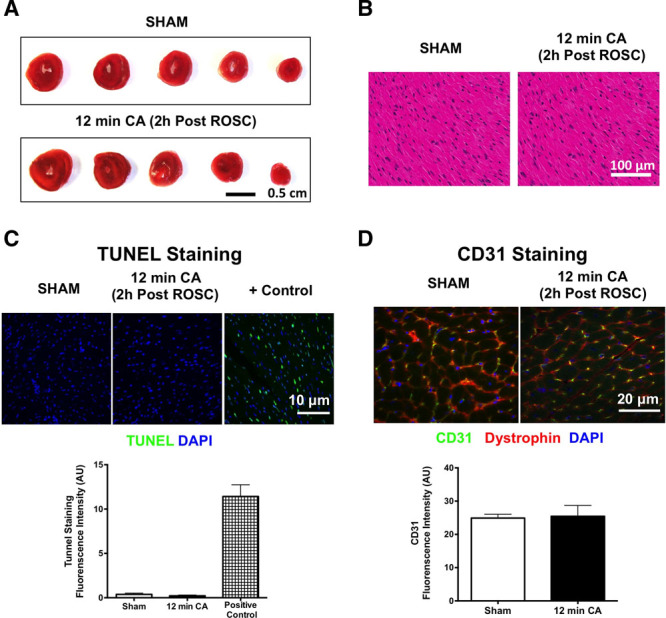
Postcardiopulmonary resuscitation myocardial dysfunction occurs in the absence of myocardium necrosis. **A**, Tetrazolium staining of hearts 2 hr following a 12-min cardiac arrest (CA). Hematoxylin and eosin staining (**B**), terminal deoxynucleotidyl transferase dUTP nick-end labeling (TUNEL) staining (**C**), and cluster of differentiation 31 (CD31) staining (**D**) of left ventricle sections 2 hr following CA compared with sham. AU = arbitrary units, DAPI = 4′,6-diamidino-2-phenylindole, ROSC = return to spontaneous circulation.

**Figure 3.**
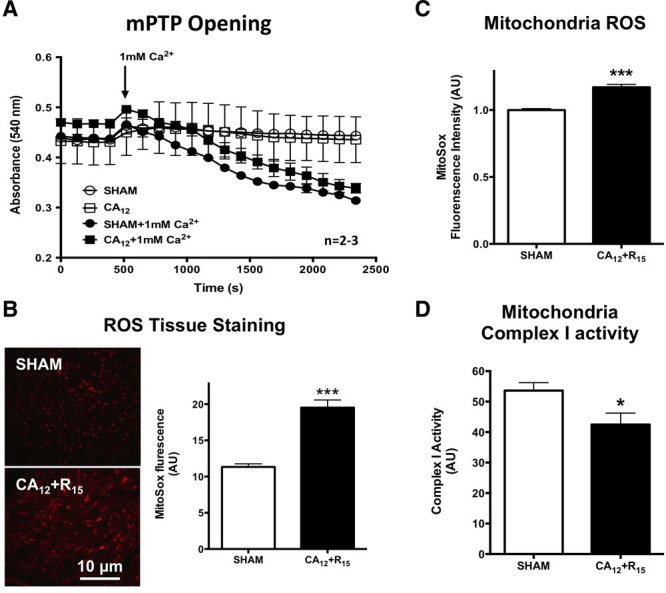
Increased reactive oxygen species (ROS) production and decreased complex I activity postcardiopulmonary resuscitation (CPR) resuscitation. **A**, Calcium-induced mitochondrial swelling from sham and post-CPR heart. *n* = 2, 3, 3, and 3, respectively. **B**, MitoSox staining (ThermoFisher, Waltham, MA) from cardiac arrest (CA) and sham mice heart. Fluorescence quantification is demonstrated in left graphic. *n* = 4, respectively. **C**, Fluorescence quantification of MitoSox staining on mitochondria isolated from CA and sham mice with the present of 10-mM pyruvate + 2-mM malate. *n* = 4, respectively. **D**, Complex I activity measurement directly from cardiac mitochondria. *n* = 4, respectively. 12 min CA (CA_12_) + reperfusion 15 min (R_15_) = 12-min CA + 15-min resuscitation; *p < 0.05; ***p < 0.001 versus sham. AU = arbitrary units, mPTP = mitochondrial permeability transition pore.

Next we sought to determine whether post-CPR myocardial dysfunction was the result of cardiomyocyte cell death. Tetrazolium staining and histologic examination revealed no evidence of myocardial necrosis (**Fig. 2**, ***A*** and ***B***), whereas terminal deoxynucleotidyl transferase dUTP nick-end labeling staining and cluster of differentiation 31 staining showed no evidence of cardiomyocyte apoptosis or endothelial cell loss (**Fig. 2, *C*** and ***D***; and **Supplemental Fig. 2**, Supplemental Digital Content 1, http://links.lww.com/CCM/F61). Increased sensitivity to mPTP opening which is associated with myocardial infarction was not observed in mitochondria isolated 15 minutes following ROSC compared with shams (**Fig. 3*A***). However, time-dependent increases in ROS were measured in post-CA tissue (**Fig. 3*B***) and mitochondria (**Fig. 3*C***) compared with shams (**Supplemental Fig. 3**, Supplemental Digital Content 1, http://links.lww.com/CCM/F61). In addition, complex I activity reduced in post-CA mitochondria compared with shams (**Fig. 3*D***). Together, these observations indicate that post-CA myocardial dysfunction is associated with post-CPR mitochondrial ROS and mitochondrial dysfunction.

### Mitochondrial Injury and Complex I and II Function Following Successful CPR

**Figure 4.**
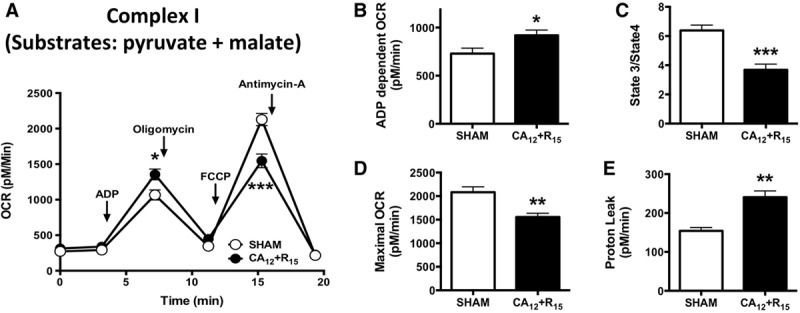
Postcardiopulmonary resuscitation mitochondrial complex I injury. Oxygen consumption rate (OCR) measurements of cardiac mitochondria from cardiac arrest (CA) and sham. **A**, The sequential injection of mitochondrial inhibitors is indicated by *arrows*. **B**, Adenosine diphosphate (ADP)-stimulated OCR. **C**, State 3/state 4 respiration. **D**, Carbonilcyanide p-triflouromethoxyphenylhydrazone-stimulated OCR. **E**, Calculated proton leak. *n* = 7, respectively. 12 min CA (CA_12_) + reperfusion 15 min (R_15_) = 12-min CA + 15-min resuscitation; *p < 0.05; **p < 0.01; ***p < 0.001 versus sham.

We next measured mitochondrial oxygen consumption in isolated mitochondria from post-CA and sham mice to further characterize post-CA mitochondrial dysfunction. Following the administration of adenosine diphosphate (ADP) to induce mitochondrial respiration, OCRs (**Fig. 4**, ***A*** and ***B***) increased as expected in both post-CPR mitochondria and sham mitochondria (919 ± 55 vs 729 ± 57 pM/min). Paradoxically, these increases were greater in the damaged post-CPR mitochondria than in the sham mitochondria but occurred in the context of increased mitochondrial proton leak (241 ± 16 vs 154 ± 9 pM/min; **Fig. 4*E***), suggesting that ADP-stimulated increases in OCR were reflective of increased ROS production rather than that of ATP production. Further evidence of post-CPR mitochondria damage was the depressed OCR observed upon maximal OCR respiration stimulated by the uncoupler carbonilcyanide p-triflouromethoxyphenylhydrazone (FCCP) (1,547 ± 97 vs 2,127 ± 86 pM/min in sham; **Fig. 4*D***) and decreases in mitochondrial efficiency of oxygen consumption based on state 3/state 4 ratios following CA compared with sham (3.7 ± 0.4 vs 6.3 ± 0.4; **Fig. 4*C***). These results are consistent with the decreased complex I activity measured directly from cardiac mitochondria after CA and further demonstrate the association of mitochondrial injury at complex I following post-CA resuscitation (Fig. 3*D*). Similar to complex I, experiments designed to measure OCR at complex II found decreases OCR in post-CPR mitochondria stimulated by FCCP compared with shams (1,554 ± 83 vs 2,082 ± 115 pM/min; **Supplemental Fig. 4*A***, Supplemental Digital Content 1, http://links.lww.com/CCM/F61). However, unlike complex I, complex II ADP-dependent OCR decreased compared with sham mitochondria (340 ± 52 vs 985 ± 74 pM/min; **Supplemental Fig. 4*B***, Supplemental Digital Content 1, http://links.lww.com/CCM/F61) with no significant differences in proton leak (998 ± 93 vs 838 ± 76 pM/min; Fig. 4*E*). These experiments demonstrate that mitochondrial injury occurs following CA resuscitation and that complex I injury differs fundamentally from complex II injury suggesting increased ROS production from this site.

### Inhibition of Complex I–Specific Superoxide Generation Reduces Myocardial Stunning and Improves Post-CPR Survival

**Figure 5.**
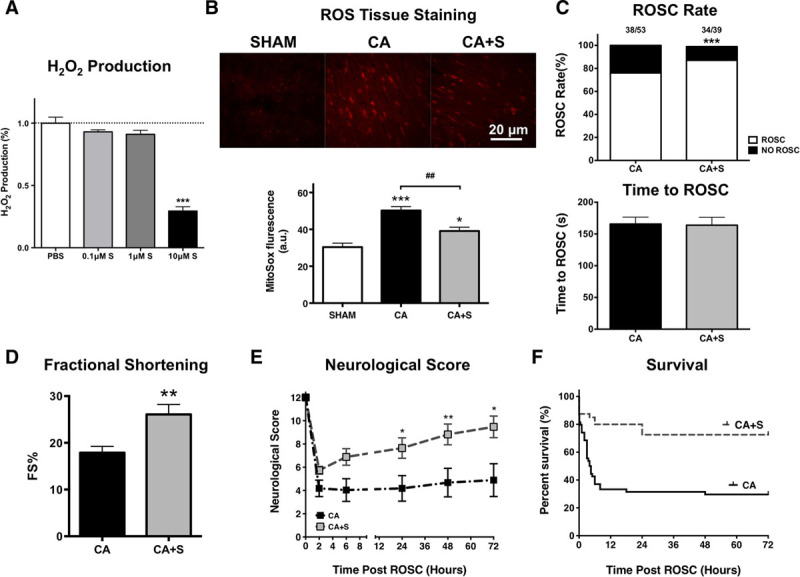
Suppressor of site I_Q_ (the ubiquinone-binding site of complex I, the active site during reverse electron transport) electron leak (S1QEL) reduces postcardiopulmonary resuscitation (CPR) myocardial stunning and improves post-CPR resuscitation outcomes. **A**, Effects of S1QEL (0.1, 1, and 10 μM) on succinate-induced H_2_O_2_ production at site I_Q_ of complex I post cardiac arrest (CA). *n* = 16, 16, 22, and 16, respectively. ***p < 0.001 versus CA group. **B**, Images and bar graph show that MitoSox staining in the heart tissue following CPR with and without S1QEL. *n* = 9, 10, and 8, respectively. *p < 0.05; ***p < 0.001 versus sham. ##*p* < 0.01 versus CA group. **C**, Return to spontaneous circulation (ROSC) following 12 min of CA and CPR time to ROSC with S1QEL and controls. *n* = 53, 39, respectively. **D**, Left ventricular fractional shortening following 12-min CA with S1QEL and controls. *n* = 10, 8, respectively. **E**, Neurologic scores in mice following CA with S1QEL and controls. *n* = 14, 17, respectively. **F**, Survival curve following CA with S1QEL and controls. S, S1QEL; *n* = 53, 39, respectively. *p < 0.05; **p < 0.01; ***p < 0.001 versus CA group. ROS = reactive oxygen species.

Because our results were indicative of increased ROS production from complex I following CA resuscitation, we next investigated whether a site-specific complex I superoxide inhibitor S1QEL would improve post-CPR outcomes. In dose-response trials, we found that a 10 μM of S1QEL was sufficient to inhibit H_2_O_2_ production in isolated mitochondria induced by 5-mM succinate at site I_Q_ (**Fig. 5*A***), while having no observable effects on cardiac function, neurological scores, or survival in normal mice (**Supplemental Fig. *5A-C***, Supplemental Digital Content 1, http://links.lww.com/CCM/F61). We next tested the effects of blinded, randomized administration of S1QEL or phosphate-buffered saline at the initiation of CPR (Supplemental Fig. 1, Supplemental Digital Content 1, http://links.lww.com/CCM/F61). Baseline animal characteristics and CPR quality were similar in both groups (**Supplemental Table 2**, Supplemental Digital Content 1, http://links.lww.com/CCM/F61). S1QEL (10 μM) reduced ROS production 15 minutes post-CA (**Fig. 5*B***) and increased the ROSC rate without altering the CPR time to ROSC (**Fig. 5*C***). S1QEL was associated with improved post-CPR myocardial contractility, neurologic function, and overall survival (fraction shortening at 2-hr post-CPR: 26% ± 2% vs 18% ± 1%; neurologic score at 72-hr post-CPR: 9.5 ± 1.0 vs 4.9 ± 1.4; survival rate at 72-hr post-CPR: 74% vs 30%; **Fig. 5*D***–***F***). The beneficial effects of S1QEL occurred in a dose-dependent manner (**Supplemental Fig. 6**, Supplemental Digital Content 1, http://links.lww.com/CCM/F61) although S1QEL did not improve the outcomes following prolonged CA (16-min CA) (**Supplemental Fig. 7**, Supplemental Digital Content 1, http://links.lww.com/CCM/F61).

## DISCUSSION

In this study, we have made “three key findings.” First, post-CPR myocardial dysfunction following asystolic CA is due to myocardial stunning rather than myocardial necrosis (Figs. 1 and 2). Although myocardial stunning is typically associated with ventricular wall movement abnormalities following brief coronary occlusion/reperfusion, our study demonstrates that stunning can occur in the context of global cardiac IR injury, which is experienced by patients resuscitated from CA. Myocardial dysfunction following induced asystolic CA has been described previously by our laboratory and others (14, 18), but in this study, we demonstrate for the first time in an asystolic CA model that post-CPR myocardial dysfunction is dependent on the length of arrest, not associated with myocardial necrosis/apoptosis, and is reversible, consistent with myocardial stunning. This stunning is similar to that previously reported in the setting of ventricular fibrillation in other animal models (8, 11). Findings of myocardial stunning described in our study and others are also consistent with reports of early recovery of myocardial function in survivors following CA in several clinical studies (23, 24). Importantly, our study demonstrates that the severity of myocardial stunning is determined by the length of CA which is related to ROSC rates and survival. Stunning is a key determinant of early post-CPR mortality and is clinically relevant. Understanding the pathophysiology of stunning is of great translational relevance in the setting of post-CA resuscitation.

Second, we discovered that post-CPR myocardial stunning occurs in the context of mitochondrial injury at complex I and II, resulting in a paradoxical increase in oxygen consumption at electron transport chain (ETC) complex I (Fig 4; and Supplemental Fig. 4, Supplemental Digital Content 1, http://links.lww.com/CCM/F61). As expected, the reduced maximal OCR at complex I and II and the decreased complex I activity were observed, supporting the finding of complex I injury following CA (Fig. 3*D*). Mitochondrial injury following post-CA resuscitation has been reported previously (25), but our unexpected observations of increased OCR with ADP administration and increased proton leak at complex I suggest that complex I could be the site of increased ROS in post-CPR ventricular tissue and mitochondria. These observations are consistent with prior reports of complex I injury associated with increased oxygen consumption and ROS generation after prolonged cardiac ischemia-reperfusion (18, 26, 27). The ROS generated following CPR in our study was not sufficient to generate opening of the mPTP but could be responsible for the observed post-CPR myocardial dysfunction given that superoxide has been demonstrated to reduce myocardial filament contractile activity in vitro in a dose-responsive manner (28).

Third, we determined that S1QEL, a site I_Q_-specific H_2_O_2_ production suppressor, limited ROS generation and neurologic injury while improving ROSC rate, myocardial function, and survival following CA (Fig. 5; and Supplemental Fig. 6, Supplemental Digital Content 1, http://links.lww.com/CCM/F61). It is well known that myocardial IR injury increases ROS generation and that targeting complex I–mediated ROS generation during reperfusion has therapeutic utility (9, 17, 18). However, a major limitation of these approaches is that they not only reduce ROS production but also limit electron flow through ETC thus disrupting normal mitochondrial function to a significant degree. Brand et al (16) have shown S1QEL overcomes these limitations and has protective effects against oxidative damage, endoplasmic reticulum stress and IR injury in the isolated perfused heart in a Langendorff preparation. To our knowledge, S1QEL has not been studied previously in vivo in mammals. Here, we show that S1QEL improves post-CPR mitochondria function resulting in reduced ROS generation and improved cardiac, neurologic, and survival outcomes in a mouse CA model. Our work has translational significance because S1QEL was administered at the time of CPR initiation and limited the effects of reperfusion injury following CA. Future research into agents that can be administered to patients by paramedics in the field to limit post-CPR reperfusion injury could represent a major advance in the caring of post-CA patients.

Our study has several limitations. First, our study was performed in a murine model of asystolic CA. Although this model has several advantages, including the ability to perform survival outcome studies and cost, our findings on the efficacy of S1QEL on myocardial function could benefit from study in other models of CA. Second, our study was not designed to determine the mechanism of S1QEL’s neuroprotective effects. It is possible that S1QEL could have had direct effects on brain ischemia-reperfusion injury although it is unknown if it is able to cross blood-brain barrier. Additional experiments will be needed to address the effects of S1QEL specifically on post-CPR neurologic injury.

## SUMMARY

In conclusion, post-CPR cardiogenic shock reflects ischemia/reperfusion-induced myocardial stunning, the severity of which depends upon the length of cardiac standstill prior to CPR. This stunning can occur following asystolic CA or following arrhythmogenic-induced CA (8, 11). Myocardial stunning is associated with a pattern of mitochondrial injury indicative of increased mitochondrial ROS generation at complex I. Targeting mitochondrial complex I ROS in the setting of post-CPR with specific inhibitors of electron leak (S1QEL) represents a novel, practical strategy to improve post-CPR resuscitation outcomes.

## ACKNOWLEDGMENTS

We thank Dr. Yun Fang for kindly helping us on measuring mitochondrial permeability transition pore opening.

## Supplementary Material

## References

[1] Benjamin EJ Virani SS Callaway CW; American Heart Association Council on Epidemiology and Prevention Statistics Committee and Stroke Statistics Subcommittee: Heart disease and stroke statistics-2018 update: A report from the American Heart Association. Circulation 2018; 137:e67–e492 29386200 10.1161/CIR.0000000000000558

[2] Larsen MP Eisenberg MS Cummins RO Predicting survival from out-of-hospital cardiac arrest: A graphic model. Ann Emerg Med 1993; 22:1652–1658 8214853 10.1016/s0196-0644(05)81302-2

[3] Kleinman ME Brennan EE Goldberger ZD Part 5: Adult basic life support and cardiopulmonary resuscitation quality: 2015 American Heart Association guidelines update for cardiopulmonary resuscitation and emergency cardiovascular care. Circulation 2015; 132:S414–S435 26472993 10.1161/CIR.0000000000000259

[4] Babini G Grassi L Russo I Duration of untreated cardiac arrest and clinical relevance of animal experiments: The relationship between the “No-Flow” duration and the severity of post-cardiac arrest syndrome in a porcine model. Shock 2018; 49:205–212 28562475 10.1097/SHK.0000000000000914

[5] Roberts BW Kilgannon JH Chansky ME Multiple organ dysfunction after return of spontaneous circulation in postcardiac arrest syndrome. Crit Care Med 2013; 41:1492–1501 23507719 10.1097/CCM.0b013e31828a39e9

[6] Nolan JP Neumar RW Adrie C Post-cardiac arrest syndrome: Epidemiology, pathophysiology, treatment, and prognostication. A scientific statement from the international liaison committee on resuscitation; the American Heart Association emergency cardiovascular care committee; the council on cardiovascular surgery and anesthesia; the council on cardiopulmonary, perioperative, and critical care; the council on clinical cardiology; the council on stroke. Resuscitation 2008; 79:350–379 18963350 10.1016/j.resuscitation.2008.09.017

[7] Braunwald E Kloner RA The stunned myocardium: Prolonged, postischemic ventricular dysfunction. Circulation 1982; 66:1146–1149 6754130 10.1161/01.cir.66.6.1146

[8] Kern KB Hilwig RW Rhee KH Myocardial dysfunction after resuscitation from cardiac arrest: An example of global myocardial stunning. J Am Coll Cardiol 1996; 28:232–240 8752819 10.1016/0735-1097(96)00130-1

[9] Bolli R Mechanism of myocardial “stunning.” Circulation 1990; 82:723–738 2203553 10.1161/01.cir.82.3.723

[10] Hirschl RB Heiss KF Bartlett RH Severe myocardial dysfunction during extracorporeal membrane oxygenation. J Pediatr Surg 1992; 27:48–53 1552444 10.1016/0022-3468(92)90103-e

[11] Yang L Li C Gao C Investigation of myocardial stunning after cardiopulmonary resuscitation in pigs. Biomed Environ Sci 2011; 24:155–162 21565687 10.3967/0895-3988.2011.02.010

[12] Abel ED Mitochondrial dynamics and metabolic regulation in cardiac and skeletal muscle. Trans Am Clin Climatol Assoc 2018; 129:266–278 30166722 PMC6116613

[13] Song M Mihara K Chen Y Mitochondrial fission and fusion factors reciprocally orchestrate mitophagic culling in mouse hearts and cultured fibroblasts. Cell Metab 2015; 21:273–286 25600785 10.1016/j.cmet.2014.12.011 PMC4318753

[14] Sharp WW Beiser DG Fang YH Inhibition of the mitochondrial fission protein dynamin-related protein 1 improves survival in a murine cardiac arrest model. Crit Care Med 2015; 43:e38–e47 25599491 10.1097/CCM.0000000000000817 PMC4342059

[15] Chouchani ET Pell VR Gaude E Ischaemic accumulation of succinate controls reperfusion injury through mitochondrial ROS. Nature 2014; 515:431–435 25383517 10.1038/nature13909 PMC4255242

[16] Brand MD Goncalves RL Orr AL Suppressors of superoxide-H2O2 production at site IQ of mitochondrial complex I protect against stem cell hyperplasia and ischemia-reperfusion injury. Cell Metab 2016; 24:582–592 27667666 10.1016/j.cmet.2016.08.012 PMC5061631

[17] Vanden Hoek TL Shao Z Li C Reperfusion injury on cardiac myocytes after simulated ischemia. Am J Physiol 1996; 270:H1334–H1341 8967373 10.1152/ajpheart.1996.270.4.H1334

[18] Dezfulian C Shiva S Alekseyenko A Nitrite therapy after cardiac arrest reduces reactive oxygen species generation, improves cardiac and neurological function, and enhances survival via reversible inhibition of mitochondrial complex I. Circulation 2009; 120:897–905 19704094 10.1161/CIRCULATIONAHA.109.853267 PMC2755623

[19] Chouchani ET Methner C Nadtochiy SM Cardioprotection by S-nitrosation of a cysteine switch on mitochondrial complex I. Nat Med 2013; 19:753–759 23708290 10.1038/nm.3212 PMC4019998

[20] Zhao D Abella BS Beiser DG Intra-arrest cooling with delayed reperfusion yields higher survival than earlier normothermic resuscitation in a mouse model of cardiac arrest. Resuscitation 2008; 77:242–249 18096292 10.1016/j.resuscitation.2007.10.015 PMC2391241

[21] Piao L Sidhu VK Fang YH FOXO1-mediated upregulation of pyruvate dehydrogenase kinase-4 (PDK4) decreases glucose oxidation and impairs right ventricular function in pulmonary hypertension: Therapeutic benefits of dichloroacetate. J Mol Med (Berl) 2013; 91:333–346 23247844 10.1007/s00109-012-0982-0 PMC3584201

[22] Jentzer JC Anavekar NS Mankad SV Changes in left ventricular systolic and diastolic function on serial echocardiography after out-of-hospital cardiac arrest. Resuscitation 2018; 126:1–6 29438721 10.1016/j.resuscitation.2018.01.050

[23] Laurent I Monchi M Chiche JD Reversible myocardial dysfunction in survivors of out-of-hospital cardiac arrest. J Am Coll Cardiol 2002; 40:2110–2116 12505221 10.1016/s0735-1097(02)02594-9

[24] Chang WT Ma MH Chien KL Postresuscitation myocardial dysfunction: Correlated factors and prognostic implications. Intensive Care Med 2007; 33:88–95 17106656 10.1007/s00134-006-0442-9

[25] Dezfulian C Kenny E Lamade A Mechanistic characterization of nitrite-mediated neuroprotection after experimental cardiac arrest. J Neurochem 2016; 139:419–431 27507435 10.1111/jnc.13764 PMC5247267

[26] Gorenkova N Robinson E Grieve DJ Conformational change of mitochondrial complex I increases ROS sensitivity during ischemia. Antioxid Redox Signal 2013; 19:1459–1468 23419200 10.1089/ars.2012.4698 PMC3797456

[27] Paradies G Petrosillo G Pistolese M Decrease in mitochondrial complex I activity in ischemic/reperfused rat heart: Involvement of reactive oxygen species and cardiolipin. Circ Res 2004; 94:53–59 14656928 10.1161/01.RES.0000109416.56608.64

[28] MacFarlane NG Miller DJ Depression of peak force without altering calcium sensitivity by the superoxide anion in chemically skinned cardiac muscle of rat. Circ Res 1992; 70:1217–1224 1315636 10.1161/01.res.70.6.1217

