# Supplementary material for: Suppression of Superoxide-Hydrogen Peroxide Production at Site IQ of Mitochondrial Complex I Attenuates Myocardial Stunning and Improves Postcardiac Arrest Outcomes
Source: Crit Care Med. 2020 Jan 15;48(2):e133–40. doi: 10.1097/CCM.0000000000004095 (PMC6964871; doi:10.1097/CCM.0000000000004095)
Supplement: Supplementary file 1 [file ccm-48-e133-s001.docx]

**Supplemental Materials:**

**Suppression of Superoxide-Hydrogen Peroxide Production at Site I_Q_ of Mitochondrial Complex I Attenuates Myocardial Stunning and Improves Post – Cardiac Arrest Outcomes**

Lin Piao, PhD^1^; Yong-Hu Fang, MD, PhD^1^_;_ Robert B. Hamanaka, PhD^2^; Gökhan M. Mutlu, MD^2^; Cameron Dezfulian, MD^3^; Stephen L Archer, MD^4^; Willard W. Sharp, MD, PhD^1^

**^1^**Section of Emergency Medicine, Department of Medicine, University of Chicago, USA

**^2^**Section of Pulmonary and Critical Care Medicine, Department of Medicine, University of Chicago, USA
^3^Safar Center for Resuscitation Research, Critical Care Medicine Department, University of Pittsburgh School of Medicine, USA
^4^Department of Medicine, Queen’s University, Canada

### Materials and Methods:

### *Cardiac arrest mice model*

Adult (age 6–8 months, 20–30g) retired breeder female C57BL/6 mice were anesthetized with 3% vaporized isoflurane and intubated/ventilated as previously described (1). Our work was based on the original work of Abella *et al.* (2) which used retired female breeders. Cardiac arrest typically affects older adults, so our study and others have used older mice to reflect this patient population. In addition, used female mice are easy to obtain from animal suppliers since they are kept for longer periods of time for breeding purposes. Because there are no observed differences between men and women regarding outcomes following sudden cardiac death, this approach has been used by cardiac arrest researchers utilizing aged mice. Asystolic CA was induced by an intravenous bolus of 0.08mg/g KCl via a jugular vein catheter and the ventilator was disconnected. Following 4, 8, 12, or 16 minutes of CA, the ventilator was reconnected, and manual chest compression were performed at a rate 350~400bpm. After 90 seconds of cardiopulmonary resuscitation (CPR), 1.5 μg of epinephrine was injected. CPR was terminated when ROSC was achieved (defined by a sinus rhythm with a mean arterial pressure greater than 40 mmHg lasting at least 5 minutes) or after 5 minutes of unsuccessful CPR. Resuscitated animals received intravenous 0.9% saline at a rate of 100 µl/h and were monitored on mechanical ventilation for up to 120 minutes. Animals used in survival studies or for observation of neurological function were returned to the animal facility and monitored. All chemicals were purchased from Sigma (St Louis, MO) unless otherwise specified. Suppressor of site I_Q_ electron leak (S1QEL), is able to reduce superoxide-H_2_O_2_ production from site I_Q_ by 40-85% at a dose of 10 μM (3). S1QEL or PBS was given to mice along with the injection of epinephrine in a blinded fashion after 90 seconds of CPR. An illustration of the CA protocol used this study was provided in **Supplemental Figure 1**.

**Mouse Echocardiography**

M-mode echocardiography was performed to monitor the cardiac function on the mice anesthetized with 3% vaporized isoflurane. Mice were secured to a Vevo 2100 (VisualSonics, Toronto, ON, Canada) platform and monitored for temperature, heart rate, and electrocardiogram as previously described (1). Transthoracic echocardiography was performed using a parasternal long-axis approach to obtain 2D left ventricular images. M-mode images were used to measure left ventricular end-diastolic and end-systolic size, and to calculate the percent fractional shortening (FS%).

**Hematoxylin and Eosin (H&E) staining**

H&E staining was prepared by University of Chicago Human Tissue Research Center. Histopathological changes in paraffin embedded hearts were examined by doing whole slide scan.

**CD31 Staining**

Frozen sections (7 μm) of mouse heart were fixed in methanol, blocked with albumin (Sigma, St. Louis, MO), and incubated with primary antibodies CD31 and Dystrophin for 1 hour at 25°C (mouse monoclonal anti-dystrophin, 1:1000 dilution, rabbit polyclonal anti-CD31, 1:500). Immunostaining was performed using standard procedures (4). Images were obtained with 3I Marianas Yokogawa-type spinning disk confocal system (Yokogawa, Tokyo, Japan) and capillary densities were analyzed by using ImageJ (NIH, Bethesda, MD).

**Tetrazolium Staining**

Tetrazolium staining of the mouse myocardial tissue has been descripted previously (5). The hearts were freshly taken and underwent a freeze-thaw cycle by wrapping heart with a clean food wrap. Hearts were then kept at -20°C for 1-2h until use. Once the hearts were solid, they were cut into 3mm slices. The slices were incubated in 1% tetrazolium salt solution stain at 37°C for 15-20min. Heart slices were then washed in PBS and images were taken under natural light.

**Terminal deoxynucleotidyl transferase mediated biotin nick end labeling (TUNEL) assay**

The myocardium apoptosis was assessed by using TACS 2-TdT Blue Apoptosis Detection Kit (Trevigen, Inc., MD) according to the manufacturer’s instructions and as previously described (6). Briefly, frozen sections (7 μm) of mouse heart were fixed with 3.7% formaldehyde in PBS for 10 min and then incubated in proteinase K at room temperature for 20 min. The sections were incubated with labeling buffer for 5 min, followed by 60 min of incubation at 37 °C in labeling reaction mix containing dNTP, TdT enzyme and CoCl_2_ and labeling buffer. The positive control was created by incubate the control slide into 1:50 TACS Nuclease buffer. The slides were then mounted with Prolong Gold antifade mounting medium (Life Technologies, Eugene, OR).

***Mitochondrial ROS measurement (MitoSox staining)***ROS measurements were made as described previously (1). Briefly, the heart sections (10 μm) were cut on a cryostat, mounted on glass slides, and stored at −70°C. At time of mitochondrial ROS measurement, slides were thawed, washed in PBS, and stained with 5 μM MitoSox in the dark for 20 min. After staining, slides were washed in PBS and imaged immediately on a Zeiss fluorescent microscope. With the addition of 10mM pyruvate + 2mM malate, Mitosox staining on isolated mitochondria was measured after 15 minutes incubation in 5 μM MitoSox in the dark for 20 minutes. The mitochondrial ROS production was quantified by measuring red fluorescence (485/530 nm) and analyzed by using ImageJ (NIH, Bethesda, MD).

***Mitochondrial permeability transition pore opening***Cardiac mitochondria (250 μg/ml) were suspended in 200 μl reaction buffer contained 120 mM KCl, 10 mM Tris (pH 7.6) and 5 mM KH_2_PO_4_ and stimulated by the addition of 1 mM CaCl_2_. The absorbance was continuously measured using a Cytation 3 (BioTek, Winooski, VT, USA) 96 well plate reader at 540 nm (7).

***Seahorse measurement of mitochondrial oxygen consumption***

The measurement procedure included four injections (ADP, oligomycin, FCCP and rotenone). Baseline OCR and stimulated (with 400 μM ADP) OCR were measured and indicated as state 2 and state 3, respectively. Following ADP depletion, oligomycin (4 μM) was added. State 4 is the OCR after the addition of oligomycin, indicating the ATP-independent respiration. Carbonyl cyanide 4-(trifluoromethoxy) phenylhydrazone (FCCP, 2 μM) was added to measure maximal uncoupled respiration and rotenone (1µM) was used to measure the proton leak. Calculated proton leak estimated by subtracting difference between oligomycin induced OCR from antimycin OCR measurements in Sham vs CA mitochondria.

**Supplemental Table 1: Pre cardiac arrest baseline characteristics of mice with 4-minute, 8-minute, 12-minute and 16-minute cardiac arrest.**

| Parameters | Sham (n=8) | CA4  (n=8) | CA8 (n=20) | CA12 (n=20) | CA16 (n=8) | *P* Value |
| --- | --- | --- | --- | --- | --- | --- |
| Body weight (g) | 26.9±0.52 | 27.0±0.46 | 27.1±0.27 | 27.4±0.40 | 27.4±0.40 | *P*>0.05 |
| Heart Rate (bpm) | 532±27.6 | 531±27.9 | 535±15.4 | 535±16.1 | 529±31.4 | *P*>0.05 |
| CPR rate (bpm) | 350±4.5 | 357.3±4.0 | 347.2±5.0 | 342.7±6.8 | 355.5±2.5 | *P*>0.05 |

Values are means±SE; n, number of animals.

**Supplemental Figure 1:**

**
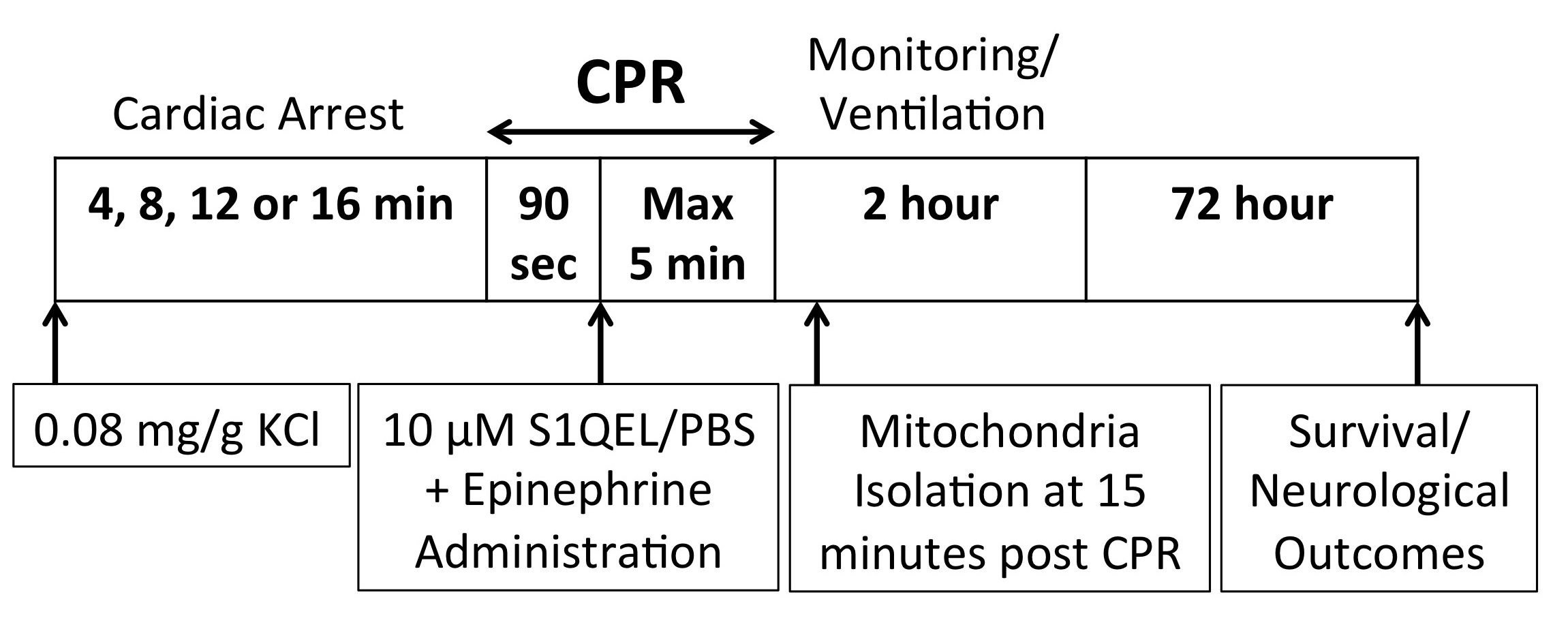
**

**Schematic of Experimental Protocol**

Six to eight-month-old female C57B6 mice were subjected to an asystolic, non-ventilated cardiac arrest induced by KCl. Ventilations and chest compressions were then performed for 90 seconds followed by intravenous epinephrine with or without S1QEL or PBS administration. CPR was then continued until ROSC or terminated after five minutes. Mice achieving ROSC were monitored and ventilated for two hours. Survival and neurological outcomes were then monitored at 72 hours.

**Supplemental Figure 2:**


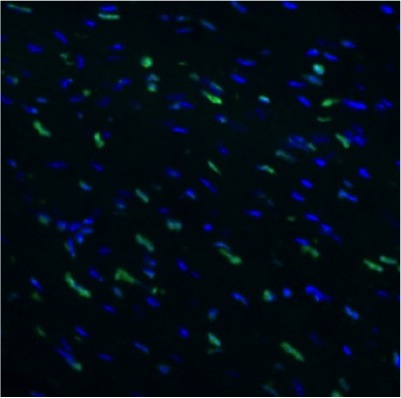


**+Control**

**SHAM**

**10 μm**

**16 min CA
(72h Post ROSC)**


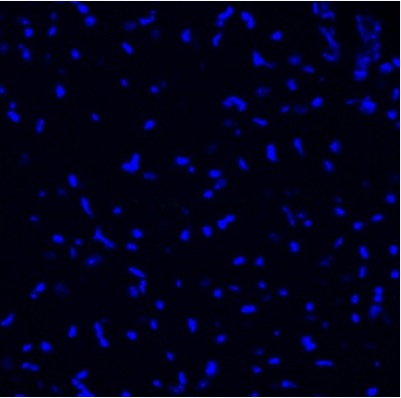

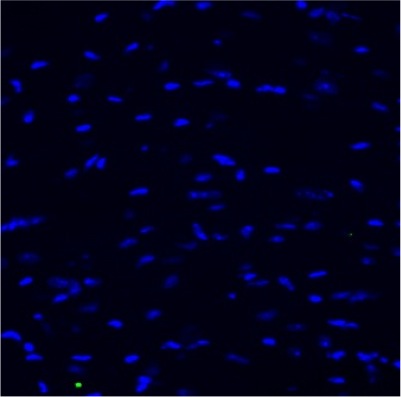


**TUNEL Staining**

**Post-CPR myocardial dysfunction occurs in the absence of myocardium apoptosis.** TUNEL staining of left ventricle at 72 hours following a 16 min CA compared to Sham and positive control.

**Supplemental Figure 3:**


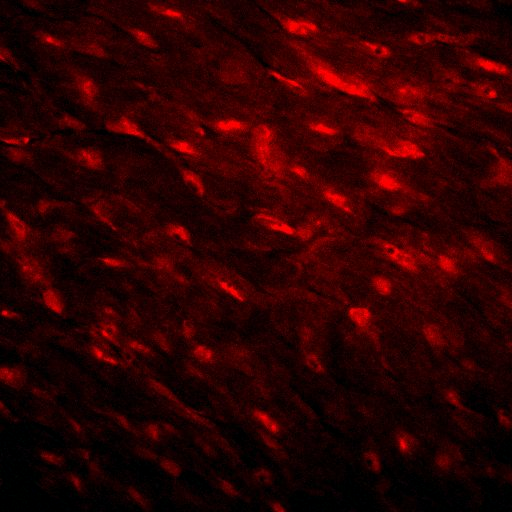

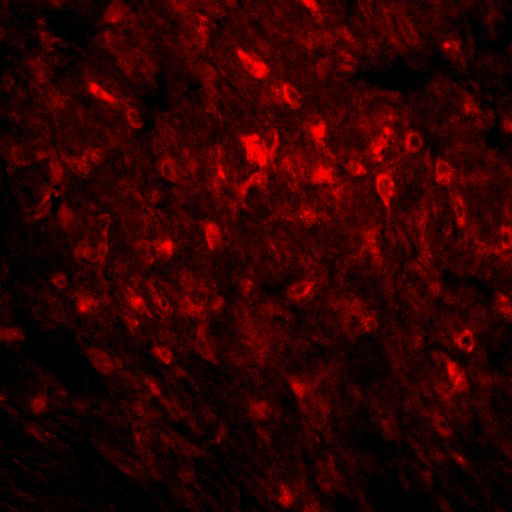

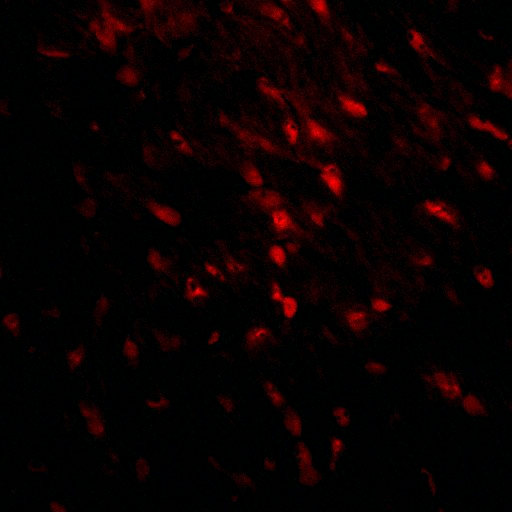

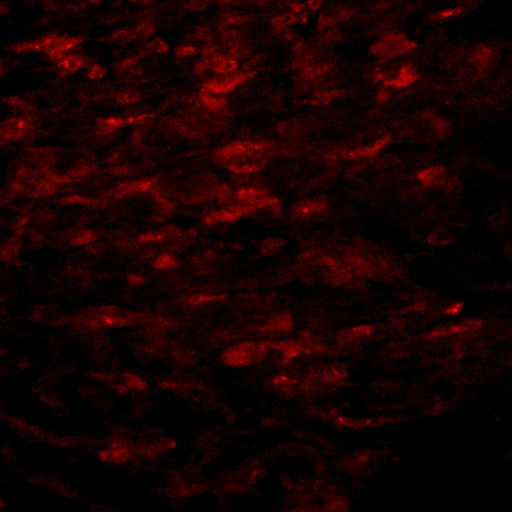


**20 μm**

**Sham**

**4min-CA**

**8min-CA**

**12min-CA**

**Post-CPR ROS production increases as CA duration is prolonged.**MitoSox staining of left ventricle tissue sections from 4-minute, 8-minute, 12-minute CA and Sham mice.

**Supplemental Figure 4:**


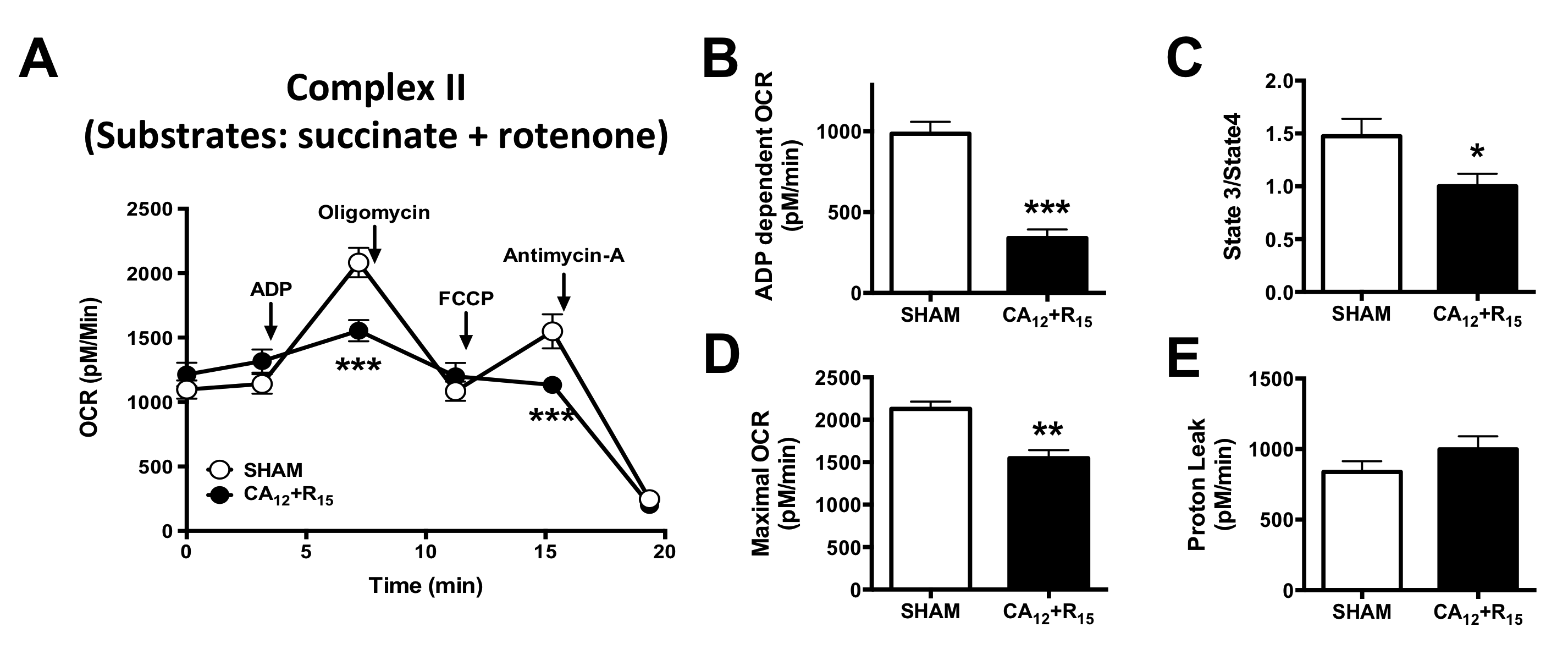


**Post-CPR mitochondrial complex II injury**

Mitochondrial complex II OCR measured using the complex I inhibitor rotenone and the substrate succinate. The sequential injection of mitochondrial inhibitors is indicated by arrows (**A**). Bar graphs show ADP induced OCR (**B**), State 3/State 4 (**C**), maximal OCR following FCCP (**D**) and proton leak (**E**). n=7, respectively. *, *P*<0.05; **, *P*<0.01; ***, *P*<0.001 vs Sham.

**Supplemental Table 2: Pre-cardiac arrest baseline characteristics of mice with and without the treatment of S1QEL.**

| Parameters | CA12 (n=40) | CA12+S1QEL (n=40) | *P* Value |
| --- | --- | --- | --- |
| Body weight (g) | 27.2±0.29 | 27.1±0.28 | *P*>0.05 |
| Heart Rate (bpm) | 534±10.9 | 540±11.4 | *P*>0.05 |
| CPR rate (bpm) | 346.4±4.2 | 346.2±4.1 | *P*>0.05 |
| Values are means±SE; n, number of animals. | | |  |

**Supplemental Figure 5:**

**A**

**B**

**C**

**S1QEL doesn’t alter cardiac function, neurological function or survival of normal mice.**S1QEL **(**10 μM) had no effect on FS% (A), neurological score (B) or survival (C) of normal mice. n=6 in each group. *P*>0.05 vs Sham.

**Supplemental Figure 6:**

**B**

**A**

**Fractional Shortening**

**Neurological Score**

**S1QEL improves cardiac and neurological functions post CPR resuscitation in a dose dependent manner.**

(**A**) Percent left ventricular fractional shortening (FS) following 12 min cardiac arrest with S1QEL (0.1 μM, 0.6 μM, 6 μM and 10 μM) and controls. (**C**) Neurological scores in mice following cardiac arrest with S1QEL and controls. n=6, 6, 8, 8 and 10, respectively. ***, *P*<0.001 vs cardiac arrest group.

**Supplemental Figure 7:**

**B**

**A**

**D**

**C**

**S1QEL had no effect on ROSC rate (A), time to ROSC (B), FS%(C) or neurological function (D) following 16-min cardiac arrest.** n=10 in each group. *P*>0.05 vs 16-min cardiac arrest group.

**References**

1. Sharp WW, Beiser DG, Fang YH, et al: Inhibition of the mitochondrial fission protein dynamin-related protein 1 improves survival in a murine cardiac arrest model. *Crit Care Med* 2015, 43(2):e38-47.

2. Abella BS, Zhao D, Alvarado J et al. Intra-arrest cooling improves outcomes in a murine cardiac arrest model. Circulation. 2004, 109(22):2786-91.

*3.* Brand MD, Goncalves RL, Orr AL, et al: Suppressors of superoxide-H_2_O_2_ production at site I_Q_ of mitochondrial complex I rrotect against stem cell hyperplasia and ischemia-reperfusion injury. *Cell Metab* 2016, 24(4):582-592.

4. Zhao D, Abella BS, Beiser DG, et al: Intra-arrest cooling with delayed reperfusion yields higher survival than earlier normothermic resuscitation in a mouse model of cardiac arrest. *Resuscitation* 2008, 77(2):242-249.

5. Ytrehus K, Liu Y, Tsuchida A, et al: Rat and rabbit heart infarction: effects of anesthesia, perfusate, risk zone, and method of infarct sizing. *Am J Physiol* 1994, 267(6 Pt 2):H2383-2390.

6. Sharp WW, Fang YH, Han M, et al: Dynamin-related protein 1 (Drp1)-mediated diastolic dysfunction in myocardial ischemia-reperfusion injury: therapeutic benefits of Drp1 inhibition to reduce mitochondrial fission. *FASEB J* 2014, 28(1):316-326.

7. Song M, Mihara K, Chen Y, S et al: Mitochondrial fission and fusion factors reciprocally orchestrate mitophagic culling in mouse hearts and cultured fibroblasts. *Cell Metab* 2015, 21(2):273-286.
